# Prediction of Initial CRP/Albumin Ratio on In-Hospital Mortality in Isolated Traumatic Brain Injury Patients

**DOI:** 10.3390/biomedicines12051084

**Published:** 2024-05-14

**Authors:** Michaela Friedrich, Kristin Haferkorn, Marco Stein, Eberhard Uhl, Michael Bender

**Affiliations:** 1 Department of Neurosurgery, Justus-Liebig-University Giessen, Klinikstraße 33, 35392 Giessen, Germany; kristin.haferkorn@neuro.med.uni-giessen.de (K.H.); marco-stein@neuro.med.uni-giessen.de (M.S.); eberhard.uhl@neuro.med.uni-giessen.de (E.U.); michael.bender@neuro.med.uni-giessen.de (M.B.); 2 Klinikum Aschaffenburg-Alzenau, Am Hasenkopf, 63739 Aschaffenburg, Germany

**Keywords:** C-reactive protein/albumin ratio, intensive care unit treatment, isolated traumatic brain injury, in-hospital mortality

## Abstract

The CRP/albumin ratio (CAR) is a mortality predictor in intensive care unit (ICU) patients. The aim of the current study was to investigate the ability of CAR to predict in-hospital mortality (IHM) in patients with isolated traumatic brain injury (iTBI). We performed a retrospective analysis including 200 patients with iTBI admitted to our neurosurgical intensive care unit (NICU) between September 2014 and December 2016. Serum biomarkers, demographic and radiological data, several ICU scores, and cardiopulmonary parameters were analyzed. The rate of IHM was 27.5% (55/200) and significantly associated with a higher AIS head score (*p* < 0.0001), a lower albumin level (*p* < 0.0001), and the necessity of a higher level of inspiratory oxygen fraction (*p* = 0.002). Furthermore, advanced age (odds ratio [OR] = 0.953, 95% confidence interval [CI] = 0.927–0.981, *p* = 0.001), a lower GCS score (OR = 1.347, 95% CI = 1.203–1.509, *p* < 0.0001), a higher level of lactate (OR = 0.506, 95% CI = 0.353–0.725, *p* < 0.0001), a higher CAR (OR = 0.547, 95% CI = 0.316–0.945, *p* = 0.031) and a higher norepinephrine application rate (OR = 0.000, 95% CI 0.000–0.090, *p* = 0.016) were identified as independent predictors of IHM. ROC analysis showed an association between IHM and a CAR cut-off value of >0.38 (Youden index 0.073, sensitivity: 27.9, specificity: 64.8, *p* = 0.044). We could identify a CAR > 0.38 as a new independent predictor for IHM in patients with iTBI.

## 1. Introduction

Traumatic brain injury is defined as an alteration in brain function or other brain pathology caused by an external force [1,2,3] and is a growing worldwide public health and socioeconomic problem [1,2,3]. Despite advancements in surgical and intensive care unit treatment, severe TBI is still associated with a high mortality rate of up to 40% [1]. Therefore, the early prognostication of outcome and in-hospital mortality is important in neurointensive care in order to advise patients and their relatives and to plan further ICU treatment. To date, the Glasgow coma scale (GCS) is mostly used for outcome prediction [4]. However, previous studies showed that the predictive value of GCS is limited [1,4,5]. Therefore, several serum biomarkers, e.g., C-reactive protein (CRP), albumin, troponin I (TNI), neuron specific enolase (NSE), and glial fibrillary acid protein (GFAP), were more frequently used for prediction of in-hospital mortality in patients with isolated traumatic brain injury (iTBI), particularly due to their cheap price and easy availability [4,6,7,8]. Several studies showed that CRP, albumin, TNI, NSE, and GFAP are associated with increased mortality in iTBI patients [4,6,7,8].

The acute-phase protein CRP follows cytokine-induced stimulation as a result of ischemia, trauma, or inflammation [9,10]. In contrast, the negative acute-phase protein albumin is downregulated in inflammation and is a well-known indicator of the current nutritional status and liver synthesis function [4,11,12,13,14,15]. Hypoalbuminemia occurs not only in patients with TBI but also in critically ill patients on ICU and patients with stroke; lower albumin levels correlate with poor outcomes and a higher mortality rate [4,9,11,12,13,15,16].

The CRP/albumin-ratio (CAR) has already been investigated as an outcome predictor in various diseases, like sepsis, cancer, cardiovascular diseases, stroke, and intracerebral hemorrhage [4,9,11,12,14,17]. It seems to have a greater predictive value than CRP alone in sepsis or septic shock, stroke, and postoperative conditions [11,13,18].

However, the predictive value of CAR for patients with TBI has not yet been sufficiently investigated. Adequate prognostic markers for TBI are indispensable for the evaluation of the patient’s current condition and the design of appropriate treatment strategies. The aim of the current study was to investigate the predictive value of CAR on in-hospital mortality in patients with iTBI [4].

## 2. Materials and Methods

### 2.1. Study Design and Population

The study protocol was approved by the Ethics Committee of Justus-Liebig University, Giessen, Germany (No. 93/20).

All patients diagnosed with iTBI admitted to the ICU of the Neurosurgical Department of the University Hospital Giessen between September 2014 and December 2016 were retrospectively analyzed. The diagnosis of iTBI was established by computed tomography (CT). The definition of iTBI comprised subdural hematoma (SDH), epidural hematoma (EDH), traumatic subarachnoid hemorrhage (tSAH), cerebral contusion, diffuse axonal injury (DAI), skull fractures, and diffuse brain injury, which was defined as a combination of the above-mentioned injury types. The exclusion criteria were as follows: (1) age of <18 years; (2) patients with injuries of other organic systems (lung, abdomen) or the musculoskeletal system (except neuro- and viscerocranium), defined as an AIS score ≥ 2; (3) patients with vascular malformations or malignant tumors as origins of the cerebral hematoma; (4) patients with spontaneous intracerebral hematoma; (5) patients with chronic SDH; (6) patients without determination of CRP and/or albumin values within the first 24 h of NICU treatment; (7) patients with acute cardiac (myocardial infarction, decompensated cardiac insufficiency) or pulmonary (pulmonary artery embolism, decompensated pulmonary hypertension) decompensation as the cause of the trauma; (8) patients with acute liver failure, chronic liver diseases, or cirrhosis; and (9) patients with infectious diseases not related to the iTBI (e.g., preexisting urinary tract infection or sepsis).

### 2.2. Data Collection

Baseline data, including age, sex, body mass index, GCS score upon admission, abbreviated injury scale (AIS) score, AIS head score, acute physiology, and chronic health evaluation II (APACHE II) score, as well as radiological, clinical, and cardiopulmonary parameters (CP) within the first 24 h of ICU treatment, were evaluated [19,20]. Blood samples, taken immediately on admission, were analyzed. Existing comorbidities (absence of comorbidities, coronary artery diseases, chronic arterial hypertension, cancer, diabetes mellitus, chronic renal insufficiency, heart failure, history of ischemic stroke or intracerebral hemorrhage (ICH)), histories of traumatic brain injury, dementia, peripheral artery disease, cardiac arrhythmia), and prehospital medications (none, beta blockers, diuretics, vitamin K antagonists, antiplatelet agents, new oral anticoagulants, antidepressants, antidiabetic and antihypertensive drugs) were extracted from the electronic medical records. Furthermore, the necessity for emergency surgery, intubation, and aspiration within the first 24 h, as well as the need for tracheostomy within inpatient treatment, were analyzed. The indication for tracheostomy was made individually by a senior resident of the ICU, depending on the cardiopulmonary state of the patient and the intracranial pressure. Tracheostomy was not usually performed before the 7th posttraumatic day. Emergency surgery included hemicraniectomy, evacuation of an epidural/subdural hematoma or a contusion hemorrhage, operative elevation of a depressed fracture, and insertion of an external ventricular drain (EVD).

### 2.3. Treatment Regimen and Intensive Care Unit Treatment

The presence of an iTBI was diagnosed by computed tomography performed in the emergency room. Patients were admitted to our neurosurgical ICU, either immediately or after emergency surgery, and treated for at least 24 h. Cardiopulmonary monitoring was performed using an invasive blood pressure measurement catheter, a pulse oximeter, and a 3-lead electrocardiogram. Additionally, a blood gas analysis was conducted every 4 h. Cardiorespiratory target parameters were defined as a systolic blood pressure of 120–140 mmHg (within the first 14 days of treatment) and an arterial oxygen partial pressure ≥ 100 mmHg. The administration of catecholamines (norepinephrine infusion) was adapted due to these systolic blood pressure target parameters. For the administration of intravenous drugs, all patients received a central venous catheter. Endotracheal intubation and pressure-controlled ventilation were performed in case of GCS ≤ 8 or respiratory insufficiency. If required, midazolam (5–40 mg/h) or propofol (200–500 mg/h) with sufentanil (35–100 µg/h) were used for analgosedation. Further medical or surgical procedures were performed based on clinical (e.g., new neurologic deficits or reduced consciousness) or radiological (e.g., rebleeding or brain swelling) impairment. Further surgical procedures included evacuation of a subdural or extradural hematoma, evacuation of a contusion hemorrhage, decompressive hemicraniectomy, insertion of an external ventricular drain (EVD), implantation of an intracranial pressure (ICP) monitoring device, operative elevation of a depressed fracture, or covering of a cerebrospinal fluid (CSF) fistula.

### 2.4. Cardiopulmonary Parameters

Cardiopulmonary parameters included mean arterial pressure, median systolic blood pressure, heart rate, respiration rate, positive end-expiratory pressure level, arterial pH value, and body temperature upon admission, as well as average norepinephrine application rate (NAR) and average inspiratory oxygen fraction (FiO_2_) during the first 24 h of ICU treatment. These data were recorded continuously or within five-minute intervals and stored in the digital ICU data recording system. Furthermore, the necessity of intubation, tracheostomy, and presence of an aspiration within the first 24 h were analyzed. Aspiration was defined as the presence of at least two of the following criteria within the first 24 h of ICU treatment: (1) detection of a radiologic correlate in either x-ray or CT scan of the thorax; (2) detection of aspiration on basis of bronchoscopy; (3) impairment of gas exchange not explained by factors other than aspiration; (4) extrahospital aspiration reported by the emergency physician; and (5) microbiologic detection of microbes in exudate of bronchus or trachea.

### 2.5. Serum Biomarkers

CRP in mg/L (ADVIA Chemistry XPT^®^ wrCRP Assay, Siemens, Munich, Germany) and albumin levels in g/L (ADVIA Chemistry XPT^®^ ALB_c Assay, Siemens, Munich, Germany) were determined directly after each patient’s admission. Further blood parameters investigated were white blood cell count in giga/L, hemoglobin value in g/dL, hematocrit value in %, platelet count in giga/L, antithrombin 3 in %, partial thromboplastin time (PTT) in seconds, prothrombin time in %, sodium in mmol/L, potassium in mmol/L, cholinesterase in U/L, blood glucose level in mg/dL, serum lactate level in mmol/L, creatinine in mg/dL, urea in mg/dL, troponin I in µg/dL, and cortisol value in µg/dL. The CRP/albumin ratio was calculated by dividing the above-mentioned albumin value into the CRP value.

### 2.6. Radiological Data

The initial CT scan of the neurocranium was analyzed in terms of the presence of an iTBI. An iTBI was defined as the presence of an SDH, an EDH, a tSAH, a contusion hemorrhage, a DAI, a skull fracture, or a diffuse iTBI. The AIS head score was also determined on the basis of the initial CT scan of the neurocranium [21]. CT analysis was performed by two independent neurosurgeons (M.F. and M.B.).

### 2.7. In-Hospital Outcome and Mortality

In-hospital outcome and mortality were evaluated using the modified Rankin scale (mRS) and the Glasgow outcome scale (GOS) at discharge [22,23]. The study population was dichotomized into survivor and non-survivor, the latter defined as mRS 6 and GOS 1, respectively.

### 2.8. Statistical Analysis

For normally distributed parameters mean ± SD was used, and for non-normally distributed parameters median/interquartile range (IQR) was used. Univariate analysis was performed with regard to all investigated parameters using the chi-square test and the Mann–Whitney U test. Subsequently, a multivariate binary logistic analysis of all univariately significant parameters was performed for the detection of an independent impact of demographic, radiological, clinical, or laboratory parameters on cardiopulmonary parameters and in-hospital mortality. Afterwards, a cutoff value for CRP/albumin ratio and GCS was calculated concerning in-hospital mortality through a Youden index and area under the curve model in a receiver operating curve (ROC). A Spearman correlation was used to investigate the correlation between NAR and CAR. All data were analyzed using an SPSS software program (IBM^®^ SPSS^®^ Statistics 24; SPSS Inc., Chicago, IL, USA).

## 3. Results

### 3.1. Main Characteristics

The total study population comprised 200 patients, with a mean age of 66.3 ± 19.9 years (range: 18–96 years). Of these, 125 patients were male (62.5%) and 75 were female (37.5%). Diffuse injury was the most frequent type of iTBI (66.5%), followed by subdural hematoma (20.5%). The median GCS at the time of admission was 11 (IQR 3–15), and the median APACHE II score was 14 (IQR 9–21). The most common comorbidity was chronic hypertension with 52%, followed by peripheral artery disease with 19.9%. In addition, the most frequent premedications were antihypertensive drugs (42%), followed by beta blockers (36.3%). Furthermore, 48.5% of iTBI patients had to be intubated and ventilated within the first 24 h, and 10% underwent tracheostomy over the course of ICU treatment. An aspiration was detected in 27% of all patients. In 46% of the patients, emergency surgery was necessary. The implementation of an EVD (22%) and the evacuation of a subdural hematoma (18%) were the most common procedures.

For maintenance of the defined cardiopulmonary target parameters, a median NAR of 0.06 µg/kg/min (IQR 0–0.04) and a median FiO_2_ of 40% (IQR 21–40) were required. The median CRP value was 2.25 mg/dL (IQR 1.1–20.7), and the median albumin values on admission were 37.5 g/L (IQR 35.1–43.1), which resulted in a median CRP/Albumin ratio of 0.067 (IQR 0.026–0.533). In Table 1, the main characteristics of the overall population are summarized.

### 3.2. In-Hospital Mortality and Outcome

Overall, the rate of in-hospital mortality was 27.5%. The in-hospital mortality for the different GCS levels is represented in Table 2. Furthermore, at the time of discharge, the median mRS was 3 (IQR 0–6) and the median GOS was 4 (IQR 1–5). A significant correlation with in-hospital mortality could be seen for advanced age (*p* = 0.002), a lower GCS score (*p* < 0.0001), a higher AIS head score (*p* < 0.0001), a higher APACHE II score (*p* < 0.0001), a higher glucose level (*p* < 0.0001), a higher lactate level (*p* < 0.0001), an elevated white blood cell count (*p* = 0.005), a higher urea level (*p* = 0.011), higher TNI level (*p* = 0.009) and an elevated CRP/albumin ratio (*p* = 0.038) at time of admission. In-hospital mortality was also associated with a lower body temperature (*p* < 0.0001), lower respiration rate (*p* < 0.0001), lower hemoglobin values (*p* = 0.001), lower hematocrit values (*p* = 0.002), reduced PTT (*p* < 0.0001), reduced prothrombin time (*p* = 0.002), a lower level of cholinesterase (*p* = 0.031) and a lower albumin level (*p* < 0.0001) at the time of admission. Non-survivors required a higher level of NAR (*p* < 0.0001) and FiO_2_ (*p* = 0.002) to maintain the defined cardiopulmonary target parameters within the first 24 h. The necessity for emergency surgery (*p* = 0.006) and intubation (*p* < 0.0001) within the first 24 h was also significantly associated with in-hospital mortality. The necessity of a tracheostomy was negatively correlated with in-hospital mortality (*p* = 0.018) (Table 3).

**Figure 1:**
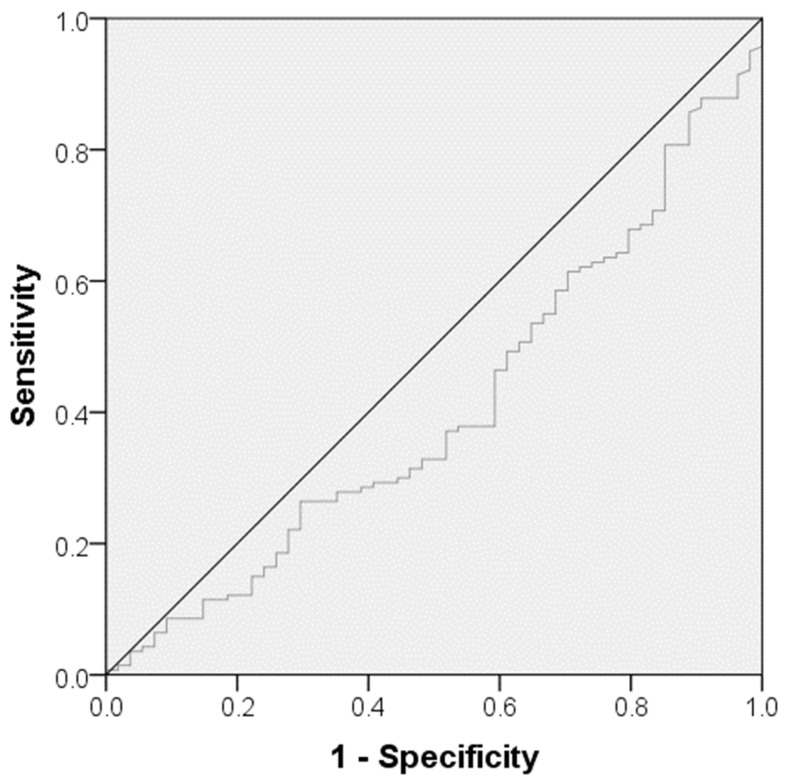
The area under the curve model in a receiver operating curve for determination of a cutoff value for CRP/albumin ratio concerning in-hospital mortality.

**Figure 2:**
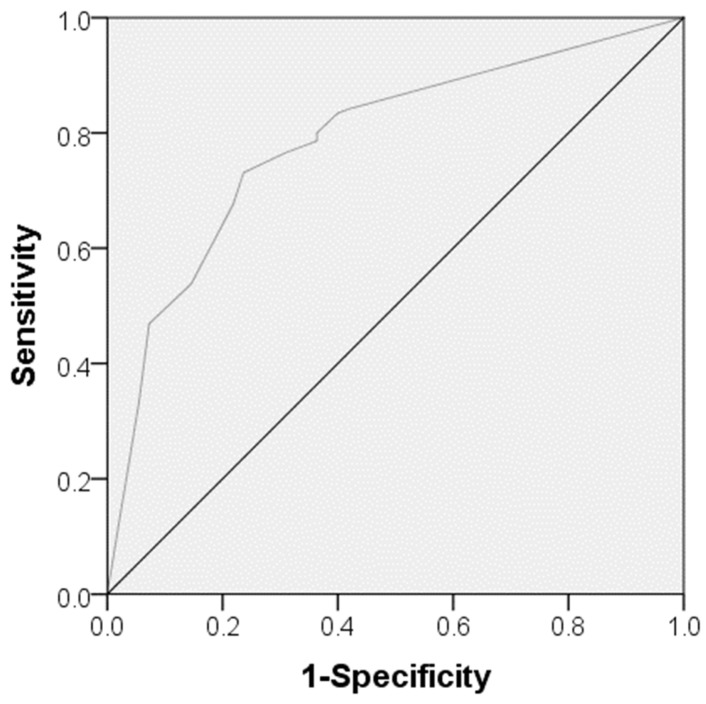
The area under the curve model in a receiver operating curve for determination of a cutoff value for the Glasgow coma scale score concerning in-hospital mortality.

**Figure 3:**
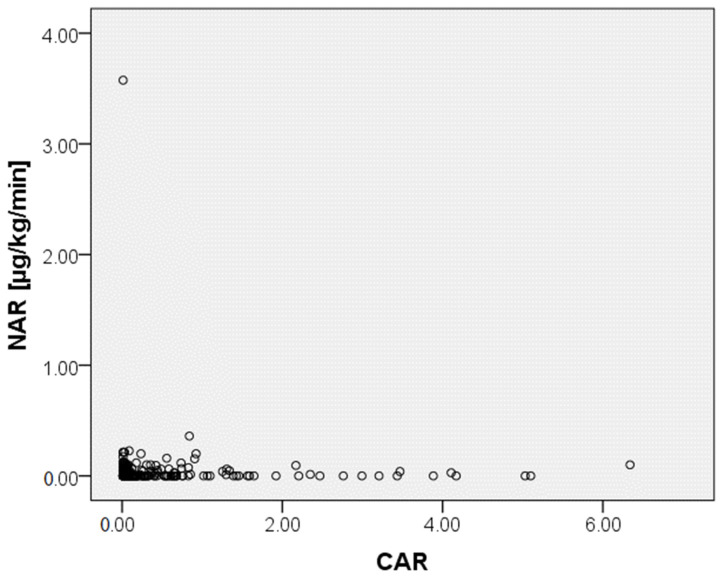
A Spearman correlation is shown for the investigation of a correlation between Norepinephrine application rate and C-reactive protein/Albumin ratio. NAR: Norepinephrine application rate, CAR: C-reactive protein/Albumin ratio; outliers are excluded here for clearer visualization.

With reference to the multivariate binary logistic analysis (Table 4), the following parameters were identified as independent predictors for in-hospital mortality at the time of admission: advanced age (odds ratio [OR] = 0.953, 95% confidence interval [CI] = 0.927–0.981, *p* = 0.001), lower GCS-Score (OR = 1.347, 95% CI = 1.203–1.509, *p* < 0.0001), a higher level of lactate (OR = 0.506, 95% CI = 0.353–0.725, *p* < 0.0001) and a higher CRP/albumin ratio (OR = 0.547, 95% CI = 0.316–0.945, *p* = 0.031) as well as a higher NAR (OR = 0.000, 95% CI 0.000–0.090, *p* = 0.016) within the first 24 h of ICU treatment. Additionally, ROC-analysis showed an association between in-hospital mortality and a CRP/albumin ratio cutoff value of >0.38 (Youden index: 0.073, sensitivity: 27.9, specificity: 64.8, *p* = 0.044) on admission (Figure 1). An association between in-hospital mortality and a GCS cutoff value of <9 on admission was determined in the dedicated ROC-analysis (Youden index: 0.49, sensitivity: 73%, specificity 76%, *p* < 0.0001) (Figure 2). The Spearman correlation between CAR and NAR showed no correlation between the two parameters (*p* = 0.237) (Figure 3).

## 4. Discussion

### 4.1. In-Hospital Mortality

This study investigated the impact of CAR on in-hospital mortality among 200 patients with iTBI admitted to our neurosurgical ICU. The rate of in-hospital mortality in our study was 27.5%, which is comparable to previous studies [1,8,24]. In addition to the well-known outcome predictor GCS, we found advanced age, higher required NAR within the first 24 h of ICU treatment, and higher levels of lactate as independent predictors for in-hospital mortality in iTBI patients. Furthermore, CAR could be identified as an independent predictor of in-hospital mortality. To date, CAR has already been proclaimed to be associated with outcome and mortality in patients with sepsis, cancer, cardiovascular diseases, stroke, and intracerebral hemorrhage [4,9,11,12,14,17]. According to our results, a CAR > 0.38 on admission is significantly associated with in-hospital mortality in iTBI patients. In addition to the known outcome parameters, CAR could be used for the estimation of the patient’s current status and the design of treatment strategies in patients with iTBI admitted to the ICU.

### 4.2. C-Reactive Protein/Albumin Ratio

The C-reactive protein (CRP) is an acute-phase protein of hepatic origin [11,14,15,17,25,26,27]. It follows a cytokine-induced stimulation after trauma, ischemia, and inflammation [9,12,28]. CRP serves as an outcome predictor for various diseases like infection, sepsis, cancer, heart failure, and postoperative conditions [4,9,11,12,13,14,25,26,29,30,31,32]. Even for ischemic stroke, intracerebral hemorrhage, and aneurysmatic subarachnoid hemorrhage, CRP is a popular outcome predictor, which is independently associated with short- and long-term mortality and functional outcome [11,31,32]. As an independent inflammation marker, CRP serves as an indicator for neuronal inflammation and secondary brain injury and is associated with the severity of cerebral trauma and mortality in TBI patients [4]. In the present study, CRP alone was not associated with in-hospital mortality in patients with iTBI. We hereby confirm previous studies, stating that CRP alone has a lower predictive value for outcome in patients with sepsis, stroke, or after surgery than CAR [11,13,18]. Xu et al. identified CRP as a prognostic biomarker for a poor 6-month outcome after TBI when measured acutely [33]. Their findings stand in contrast to our study, not identifying CRP alone as an outcome predictor for iTBI in patients with IHM [33]. This discrepancy may be due to the larger study population and the fact that we excluded patients with injuries of other organic systems or the musculoskeletal system, patients with acute cardiac or pulmonary decompensation, patients with acute liver failure, chronic liver diseases, or cirrhosis, and patients with infectious diseases not related to the iTBI. However, the most important difference between the two studies is the primary end point [33]. We investigated the predictive value of CRP for IHM, whereas Xu et al. evaluated the outcome after 6 months [33].

Albumin is a non-specific transport protein for endogenous and exogenous substances in blood plasma synthesized in the liver [4,11]. As a negative acute-phase protein, it was also investigated as an outcome predictor in critically ill patients with sepsis and septic shock [11,12,14,15,17,18,29]. It is also known for its neuroprotective properties and serves as a prognostic factor in ischemic stroke [11]. Hypalbuminemia leads to fluid transfer into brain tissue, resulting in cerebral edema and increased intracranial pressure, and is associated with poor outcomes in TBI patients [4]. We identified an association between lower albumin levels and in-hospital mortality in iTBI patients (<0.0001). However, albumin is an indicator, not just for inflammation, but also for the nutritional status and liver synthesis function, and its predictive value is susceptible to bias [9,11,29]. Therefore, the CAR was implemented as a novel ratio to avoid the potential negative aspects of the singular biomarkers CRP and albumin and combine information about the relation of systemic inflammation, dystrophia, and nutritional status in a new biomarker [4,9,11,12,13,14,27,29]. The CRP/Albumin-ratio (CAR) serves as a better indicator of the inflammatory status with a higher predictive value than CRP or Albumin alone [4,9,11]. To date, the predictive value of CAR has been investigated in patients admitted to general ICUs or in patients with stroke or intracerebral hemorrhage, but data about CAR as an outcome predictor in patients with iTBI have not yet been adequately investigated [4,9,11,12,14]. In the present study, we could identify CAR as an independent predictor for in-hospital mortality in patients with iTBI. These results align with a previous study that also identified CAR as an independent predictor for in-hospital mortality in patients with moderate to severe TBI [24]. We therefore found a cutoff value >0.38 to be associated with in-hospital mortality. Oh et al. defined a cutoff value >1.75 for 30-day mortality and a cutoff value >1.58 for 1-year mortality in patients admitted to the ICU after surgery [13]. Ranzani et al. defined a similar cutoff value >2 for estimation of long-term mortality in patients with severe sepsis or septic shock, and a cutoff value >1.22 was defined by Bender et al. for increased in-hospital mortality in patients with intracerebral hemorrhage [12,15]. Considerably different cutoff values were identified for 28-day mortality in critically ill patients admitted to ICU (>34.3) and for overall mortality in patients after emergency surgery (>40) [9,17]. Consequentially, cutoff values seem to be highly dependent on the investigated study population [13]. Furthermore, in our study and in previous studies, non-matching metrics were used for CRP (mg/dL) and albumin (g/L), making comparisons between cutoff values more challenging. Our estimated cutoff value of >0.38 may be comparatively low due to the fact that the iTBI study population at the time of admission had lower CRP and higher albumin levels in comparison to, e.g., patients suffering from sepsis, pneumonia, or cancer, or patients after excessive abdominal surgery. The discrepancy between the higher cutoff values in the literature and the low cutoff value in our study can also be attributed to variations in the study populations. Inpatient admission to general ICUs is frequently justified by an infection (e.g., pneumonia or sepsis), acute heart decompensation (e.g., heart attack or unstable coronary artery diseases), or surgery (e.g., oncological surgery). These patients have higher inflammatory markers upon admission and hence a higher CRP/albumin ratio than neurocritically ill patients, in which loss of consciousness is the main indication for ICU treatment.

Given the fact that norepinephrine is used for the maintenance of our defined cardiopulmonary parameters, it can be stated that patients receiving norepinephrine suffered from hypotension. To investigate whether there is any influence of CAR on the required norepinephrine application rate, we added the investigation into whether there is a statistical correlation between CAR and NAR. However, the Spearman correlation between CAR and NAR showed no correlation between the two parameters. Subsequently, one can act on the assumption that the impact of CAR on in-hospital mortality is not due to hypotension.

### 4.3. Limitations of the Study

This study has its strengths, but it also suffers from several limitations. First, it is a retrospective, single-center study. Furthermore, we have no repeated measurements of CRP and albumin values and therefore no follow-up CRP/albumin ratios. In addition, the findings cannot be applied to all types of iTBI due to the exclusion of patients with polytrauma, vascular malformations, or malignancy. Mostly, the estimation of in-hospital mortality for different GCS levels and the ROC study for determination of a cutoff value for the association between in-hospital mortality and GCS should be taken with caution because of the low number of cases. Taking into account that the sensitivity, specificity, and Youden index of the ROC analysis are relatively low, the estimated cutoff value must be treated with caution. CAR cannot be identified as a standalone prediction parameter of in-hospital mortality in patients with iTBI and has to be combined with other well-known prediction parameters, like GCS [4].

The strength of the current study is its large study population with comprehensive demographic characteristics, as well as clinical, radiological, and laboratory chemistry data records. In addition, to the authors’ knowledge, this is the first study to investigate the impact of CAR on the in-hospital mortality of neurosurgical ICU patients with iTBI.

There is ongoing prospective research at the University Hospital of Giessen into the predictive value of CAR concerning in-hospital mortality in iTBI patients. We are planning to improve the methodology of the prospective study concerning the limitations of the current one, as we plan a higher number of cases, a thorough exclusion of patients with preexisting illnesses influencing CRP or albumin levels, and an accurate determination of the time points of blood sampling. If the results turn out to be as promising as the retrospective ones, it will be a highly interesting topic to evaluate CAR at different time points in future studies and to investigate whether a decrease in CAR during ICU treatment changes mortality.

## 5. Conclusions

CAR was found to be a new independent predictor of in-hospital mortality in neurosurgical ICU patients with iTBI. An increased risk of in-hospital mortality was identified in iTBI patients with a CAR greater than 0.38 upon admission. Combined with other well-known outcome predictors, this ratio could be a helpful serum biomarker for early decision making with regard to initiating or declining further ICU treatment in these patients.

## Figures and Tables

**Table 1:** Main characteristics of the study population (n = 200).

Parameter	Results
**Baseline data**	
Age, years, mean (±SD) *	66.3 (19.9)
Women, n (%) *	75 (37.5)
Men, n (%) *	125 (62.5)
Body mass index, kg/m^2^, mean (±SD) *	26.0 (4.1)
Glasgow coma scale score, median (IQR) *	11 (3–15)
AIS score, median (IQR) *	0 (0–0)
AIS score 0, n (%) *	160 (80.0)
AIS score 1, n (%) *	40 (20.0)
AIS head score, median (IQR) *	2 (1–5)
AIS head score 1, n (%) *	52 (26.0)
AIS head score 2, n (%) *	55 (27.5)
AIS head score 3, n (%) *	7 (3.5)
AIS head score 4, n (%) *	22 (11.0)
AIS head score 5, n (%) *	31 (15.5)
AIS head score 6, n (%) *	33 (16.5)
APACHE II score, median (IQR) *	14 (9–21)
Comorbidities, n (%) *	130 (65.0)
Premedication, n (%) *	124 (62.0)
Emergency surgery, n (%) ***	79 (39.5)
Intubation, n (%) **	97 (48.5)
Tracheostomy, n (%) ***	20 (10.0)
Aspiration, n (%) ***	54 (27.0)
**Cardiopulmonary parameters**	
Norepinephrine application rate, µg/kg/min, median (IQR) **	0.06 (0–0.04)
Systolic blood pressure, mmHg, median (IQR) **	128 (118–138)
Mean arterial pressure, mmHg, median (IQR) **	81 (74–90)
Heart rate, beats per minute, median (IQR) **	75.5 (67.8–87.3)
Respiration rate, n per minute, median (IQR) **	12 (12–17)
Inspiratory oxygen fraction, %, median (IQR) **	40.0 (21–40)
PEEP level, median (IQR) **	6 (5–7)
Body temperature, centigrade, median (IQR) *	36.7 (36.2–37.3)
**Biomarkers**	
White blood cells, giga/L, median (IQR) **	11.8 (11.4–14.7)
Hemoglobin, g/dL, median (IQR) **	13.0 (11.4–14.5)
Hematocrit, l/L, median (IQR) **	0.38 (0.34–0.42)
Thrombocytes, giga/L, median (IQR) **	181.5 (155–261)
Prothrombin time, %, median (IQR) **	83.5 (70.5–101)
Partial thromboplastin time, sec, median (IQR) **	27.5 (26–33)
Fibrinogen, g/L, median (IQR) **	2.55 (2.31–3.35)
Antithrombin 3, %, median (IQR) **	85.5 (75.3–97.8)
Sodium, mmol/L, median (IQR) **	139 (137–141)
Potassium, mmol/L, median (IQR) **	3.9 (3.6–4.2)
Creatinine, mg/dL, median (IQR) **	0.8 (0.6–1.1)
Urea, mg/dL, median (IQR) **	28 (25.3–47.8)
Cholinesterase, U/L, median (IQR) **	7246 (5378–9255)
Blood glucose, mg/dL, median (IQR) **	134.5 (112–163)
Serum lactate, mmol/L, median (IQR) **	1.5 (1.0–2.3)
Troponin I, ng/mL, median (IQR) **	0.01 (0.01–0.04)
Cortisol, µg/dL, median (IQR) **	23.2 (16.6–36.6)
C-reactive protein, mg/dL, median (IQR) **	2.25 (1.1–20.7)
Albumin, g/L, median (IQR) **	37.5 (35.1–43.1)
C-reactive protein/Albumin ratio, median (IQR) **	0.067 (0.026–0.533)
**Radiological data**	
Subdural hematoma, n (%) *	41 (20.5)
Epidural hematoma, n (%) *	5 (2.5)
Traumatic subarachnoid hemorrhage, n (%) *	11 (5.5)
Brain contusion, n (%) *	6 (3)
Diffuse axonal injury, n (%) *	3 (1.5)
Skull fracture, n (%) *	1 (0.5)
Diffuse traumatic brain injury, n (%) *	133 (66.5)
**Outcome**	
mRS score, median (IQR) ****	3 (0–6)
mRS 0, n (%) ****	68 (34)
mRS 1, n (%) ****	13 (56.5)
mRS 2, n (%) ****	8 (4)
mRS 3, n (%) ****	18 (9)
mRS 4, n (%) ****	11 (5.5)
mRS 5, n (%) ****	27 (13.5)
mRS 6, n (%) ****	55 (27.5)
GOS score, median (IQR) ****	4 (1–5)
GOS 1, n (%) ****	55 (27.5)
GOS 2, n (%) ****	13 (6.5)
GOS 3, n (%) ****	25 (12.5)
GOS 4, n (%) ****	27 (13.5)
GOS 5, n (%) ****	80 (40)

SD: standard deviation, IQR: interquartile range, AIS: Abbreviated Injury scale, APACHE II: Acute Physiology and Chronic Health Evaluation II, PEEP: positive end expiratory pressure, mRS: modified Rankin Scale, GOS: Glasgow Outcome Scale. * upon admission, ** within the first 24 h, *** during inpatient treatment, **** at discharge.

**Table 2:** In-hospital mortality (IHM) for different Glasgow coma scale (GCS) levels.

GCS	Survivor	Non-Survivor	IHM [%]
3	23	32	58.18
4	1	1	50
5	5	2	28.57
6	2	0	0
7	3	3	50
8	5	4	44.44
9	0	0	-
10	8	1	11.11
11	10	2	16.67
12	10	2	16.67
13	10	4	28.57
14	20	1	4.76
15	48	3	5.88

**Table 3:** Univariate predictors of in-hospital mortality.

Parameter	Survivor (n = 145)	Non-Survivor (n = 55)	*p*-Value
**Baseline Data**			
Age, years, mean (±SD) *	63.5 (20.9)	73.6 (14.5)	**0.002**
Women, n (%) *	55 (37.9)	20 (36.4)	0.838
Men, n (%) *	90 (62.1)	35 (63.6)	
Body mass index, kg/m^2^, mean (±SD) *	25.9 (3.7)	26.3 (4.9)	0.397
Glasgow coma scale score, median (IQR) *	13 (8–15)	3 (3–8)	**<0.0001**
AIS score, median (IQR) *	0 (0–0)	0 (0–1)	0.113
AIS score 0, n (%) *	120 (82.8)	40 (72.7)	
AIS score 1, n (%) *	25 (17.2)	15 (27.3)	
AIS head score, median (IQR) *	2 (1–3)	6 (5–6)	**<0.0001**
AIS head score 1, n (%) *	52 (35.9)	0 (0)	
AIS head score 2, n (%) *	55 (37.9)	0 (0)	
AIS head score 3, n (%) *	7 (4.8)	0 (0)	
AIS head score 4, n (%) *	9 (6.2)	13 (23.6)	
AIS head score 5, n (%) *	22 (15.2)	9 (16.4)	
AIS head score 6, n (%) *	0 (0)	33 (60.0)	
APACHE II score, median (IQR) *	11 (8–17)	21.5 (18–26.3)	**<0.0001**
Comorbidities, n (%) *	91 (62.8)	39 (70.9)	0.407
Premedication, n (%) *	91 (62.8)	33 (60)	0.130
Emergency surgery, n (%) ***	53 (36.6)	26 (47.3)	**0.006**
Intubation, n (%) **	49 (33.8)	48 (87.3)	**<0.0001**
Tracheostomy, n (%) ***	19 (13.1)	1 (1.8)	**0.018**
Aspiration, n (%) ***	35 (24.1)	19 (34.5)	0.256
**Cardiopulmonary parameter**			
Norepinephrine application rate, µg/kg/min, median (IQR) **	0.0 (0.0–0.03)	0.03 (0.00–0.01)	**<0.0001**
Mean arterial pressure, mmHg, median (IQR) **	82 (74–90)	78.5 (67.3–91.5)	0.126
Heart rate, beats per minute, median (IQR) **	76 (68–87)	72 (63–88.5)	0.246
Respiration rate, n per minute, median (IQR) **	15 (12–17)	12 (10–15)	**<0.0001**
Inspiratory oxygen fraction, %, median (IQR) **	21 (21–40)	35 (21–45)	**0.002**
PEEP level, median (IQR) **	5.5 (5–7)	5 (5–6)	0.089
Body temperature, centigrade, median (IQR) *	36.9 (36.4–37.3)	36.3 (35.1–36.8)	**<0.0001**
**Biomarkers**			
White blood cells, giga/L, median (IQR) **	10.0 (7.4–14.3)	11.9 (9.8–15.7)	**0.005**
Hemoglobin, g/dL, median (IQR) **	13.5 (11.9–14.8)	12.2 (10–13.8)	**0.001**
Hematocrit, l/L, median (IQR) **	0.39 (0.35–0.43)	0.36 (0.3–0.4)	**0.002**
Thrombocytes, giga/L, median (IQR) **	209 (160–272.5)	193 (144–251)	0.130
Prothrombin time, %, median (IQR) **	94 (80–102)	82 (56–96)	**0.002**
Partial thromboplastin time, sec, median (IQR) **	28 (26–31)	31 (27–39)	**<0.0001**
Fibrinogen, g/L, median (IQR) **	2.77 (2.32–3.33)	2.72 (2.22–3.46)	0.793
Antithrombin 3, %, median (IQR) **	89 (77.3–97.8)	284 (71.5–98.3)	0.364
Creatinine, mg/dL, median (IQR) **	0.8 (0.6–1)	0.9 (0.7–1.3)	0.087
Urea, mg/dL, median (IQR) **	34 (24.5–46)	42 (28–59)	**0.011**
Cholinesterase, U/L, median (IQR) **	7711.5 (5757.5–9332)	6555 (3863–8942)	**0.031**
Blood glucose, mg/dL, median (IQR) **	123 (110–154)	158 (131–182)	**<0.0001**
Serum lactate, mmol/L, median (IQR) **	1.3 (0.9–2.1)	2.1 (1.2–3.2)	**<0.0001**
Troponin I, ng/mL, median (IQR) **	0.01 (0.01–0.02)	0.02 (0.01–0.08)	**0.009**
Cortisol, µg/dL, median (IQR) **	24.4 (16.4–35.9)	28 (17.6–45)	0.250
C-reactive protein, mg/dL, median (IQR) **	4.01 (0.88–19.47)	8.1 (1.6–24)	0.169
Albumin, g/L, median (IQR) **	39.5 (36.5–43.7)	36.4 (32.6–41.1)	**<0.0001**
C-reactive protein/Albumin ratio, median (IQR) **	0.09 (0.02–0.48)	0.24 (0.04–0.68)	**0.038**
**Radiological data**			
Localization			0.158
Subdural hematoma, n (%) *	27 (18.6)	14 (25.5)	
Epidural hematoma, n (%) *	5 (3.4)	0 (0)	
Traumatic subarachnoid hemorrhage, n (%) *	11 (7.6)	0 (0)	
Brain contusion, n (%) *	5 (3.4)	1 (1.8)	
Diffuse axonal injury, n (%) *	3 (2.1)	0 (0)	
Skull fracture, n (%) *	1 (0.7)	0 (0)	
Diffuse traumatic brain injury, n (%) *	93 (64.1)	40 (72.7)	

SD: standard deviation, IQR: interquartile range, AIS: Abbreviated Injury scale, APACHE II: Acute Physiology and Chronic Health Evaluation II, PEEP: positive end expiratory pressure. * upon admission, ** within the first 24 h, *** during inpatient treatment. **Bold**: *p* < 0.05.

**Table 4:** Cox regression hazard model in relation to in-hospital mortality.

Parameter	Odds Ratio	95% CI	*p*-Value
Age, years, mean (±SD) *	0.953	0.927–0.981	**0.001**
Glasgow coma scale score, median (IQR) *	1.347	1.203–1.509	**<0.0001**
Norepinephrine application rate, µg/kg/min, mean (±SD) **	0.000	0.000–0.090	**0.016**
Serum lactate, mmol/L, median (IQR) **	0.506	0.353–0.725	**<0.0001**
Inspiratory oxygen fraction, mean (±SD) **	1.041	0.995–1.088	0.079
C-reactive protein/Albumin ratio, mean (±SD) *	0.547	0.316–0.945	**0.031**

SD: standard deviation, IQR: interquartile range, * upon admission, ** within the first 24 h. **Bold**: *p* < 0.05.

## Data Availability

The original contributions presented in the study are included in the article; further inquiries can be directed to the corresponding author.
